# Ternary Polyaniline@Bi_2_O_3_-BiOCl Nanocomposites as Innovative Highly Active Photocatalysts for the Removal of the Dye under Solar Light Irradiation

**DOI:** 10.3390/nano13040713

**Published:** 2023-02-13

**Authors:** Asmae Bouziani, Mohamed Yahya, Claudia L. Bianchi, Ermelinda Falletta, Gokhan Celik

**Affiliations:** 1 Chemical Engineering Department, Middle East Technical University, 06800 Ankara, Turkey; 2 Department of Chemistry, University of Nevada, Reno 1664 North Virginia Street, Reno, NV 89557, USA; 3 Department of Chemistry, Università degli Studi di Milano, Via C. Golgi 19, 20133 Milano, Italy; 4 Consorzio Interuniversitario Nazionale per la Scienza e Tecnologia dei Materiali (INSTM), Via Giusti 9, 50121 Florence, Italy

**Keywords:** nanocomposites, polyaniline, Bi_2_O_3_, BiOCl, photocatalysis, solar light, dye

## Abstract

Ternary PANI@Bi_2_O_3_-BiOCl nanocomposites were successfully synthesized during the oxidative polymerization of aniline monomer in the presence of Bi_2_O_3_. PANI@Bi_2_O_3_-BiOCl nanocomposites were characterized by several analytical techniques, including X-ray diffraction (XRD), Fourier Transform Infrared spectroscopy (FTIR), N_2_ physisorption, UV–Vis Diffuse reflectance spectroscopy (DRS), X-ray photoelectron spectroscopy (XPS), transmission electron microscopy (TEM), and scanning electron microscopy with energy dispersive spectroscopy (SEM-EDS). The effective PANI-semiconductor interaction promotes the fast separation and transfer of photogenerated electrons and holes, enhancing the photocatalytic efficiency of the materials towards methylene blue (MB) degradation under solar light irradiation. The best results were obtained by 0.5%PANI@Bi_2_O_3_-BiOCl, leading to 80% MB degradation in 2 h, four times higher than pristine Bi_2_O_3_-BiOCl. Moreover, 0.5%PANI@Bi_2_O_3_-BiOCl maintained stable photocatalytic performances for four cycles without significant activity loss. Various scavengers (isopropyl alcohol, formic acid, and benzoquinone) were used to identify the active species by trapping holes and radicals generated during the photocatalytic degradation process. Finally, a probable photocatalytic mechanism of PANI@Bi_2_O_3_-BiOCl photocatalyst was suggested.

## 1. Introduction

Combining organic and inorganic compounds to obtain new materials displaying the properties of both components and novel performances is a well-established technique. Performing such modification by combining organic–inorganic species has extraordinary effects on the production of nano-sized materials with multifunctional applications in several fields, such as gas sensors, tissue engineering, photovoltaic cells, adsorption and photocatalysis [1,2,3]. 

Photocatalytic degradation of organic pollutants, including organic dyes, represents an important example of green technology [4,5,6,7]. This technology is based on reactive and nonselective species with a superior oxidizing capability compared to traditional ones (Cl_2_, O_3_, H_2_O_2_, …) [8,9]. Photocatalysis has drawn substantial attention after Fujishima and Honda described water splitting by photochemical electrodes using TiO_2_ [10]. Although several conventional and commercially available semiconductors (ZnO, TiO_2_, etc.) [11,12] have been extensively investigated, their limited activity under solar light represents a significant downside for transitioning from fundamental study to practical applications. Thus, for the wide use of the solar spectrum, exploring new cost-effective and stable photocatalysts with a visible light response is of particular importance and urgency [5,13,14,15,16,17,18]. Bismuth-based catalysts, such as Bi_2_O_3_, Bi_2_S_3_, and BiOCl, are important semiconductor materials that have drawn significant interest due to their catalytic activity in numerous fields and being environmentally friendly [19,20]. Unfortunately, pristine Bi_2_O_3_ has low practical application in the field of photocatalysis because of its narrow response range of visible light and the fast recombination of electron-hole pairs, as well as the inability of the electron at the conduction band (CB) to reduce oxygen (E_cb_ = 0.33 V vs. NHE) [21]. In contrast, BiOCl is an indirect bandgap semiconductor with a high anisotropic layer structure and is chemically stable. However, its bandgap energy between 3.2 and 3.5 eV makes it a traditional UV photocatalyst. Bi_2_O_3_-BiOCl heterojunctions lead to better transport and separation of photogenerated charges resulting in improved photocatalytic activity due to the efficient separation of photoinduced e^−^/h^+^ pairs [22,23].

In recent years, conducting organic properties, particularly polyaniline (PANI), have emerged as very interesting materials for several applications, including photocatalysis.

Thanks to its interesting multifunctionality and redox properties, PANI and its composites have been employed in the removal of different types of pollutants from both water and air. In fact, PANI exhibited extraordinary ability in VOCs [24] and NO**_2_** [25] abatement from the air matrix, as well as metals in the removal of metals and dyes from water [26,27,28].

In fact, when used in combination with proper semiconductors, the π–π conjugated structure of the polymer can rapidly transfer the charge on the surface of the material, enhancing the separation of the photoinduced charges and reducing the recombination rate during the electron-transfer process [29,30,31], improving, as a consequence, the photocatalytic activity.

Preparing PANI-semiconductor composites has drawn significant attention because of their particular electron and hole-transporting properties, simplicity of synthesis, and chemical stability [6,32,33,34,35]. Although the effect of PANI coating on traditional semiconductors, mainly TiO**_2_** and ZnO, has been extensively investigated, only a few studies have been dedicated to the photocatalytic properties of PANI/Bi_2_O_3_ composites. Recently, PANI@BiOCl has been synthesized and tested for the degradation of dyes under visible light irradiation, showing very promising results [34,36,37], as well as PANI@Bi_2_O_3_ [38,39]. However, for this latter material, more in-depth investigations are necessary to clarify the structure of the composite and, as a consequence, the role of the polymer in the photocatalytic activity of the heterostructure. In this work, a simple synthetic method leading to ternary nanocomposites (PANI@Bi_2_O_3_-BiOCl) characterized by enhanced photocatalytic properties is proposed. For the first time, it has been demonstrated the formation of a second Bi(III) phase (BiOCl) during the oxidative polymerization of aniline on the surface of Bi_2_O_3_. The synthesized nanocomposites were tested for the photodegradation of Methyl Blue (MB), selected as a model non-biodegradable and toxic molecule, under solar light irradiation. The reusability of the more performing photocatalytic nanocomposite has been adequately investigated, indicating high stability for up to five runs. Eventually, the active species involved in the photocatalytic process were properly identified using various scavengers’ trapping holes and radicals.

## 2. Materials and Methods

### 2.1. Chemicals

The chemicals utilized to synthesize Bi_2_O_3_ nanoparticles were bismuth(III) nitrate pentahydrate (Bi(NO_3_)_3_·5H_2_O), nitric acid (HNO_3_, 70%), and citric acid (C_6_H_8_O_7_, 99.5%). In addition, aniline monomer (C_6_H_8_N), hydrochloric acid (HCl, 37%), and sodium persulfate (Na_2_S_2_O_8_) were employed for polyaniline (PANI) synthesis. They were obtained from Sigma-Aldrich (Merck & Co., St. Louis, MO, USA).

Methylene Blue powder (MB, 97%), selected as organic pollutants for photocatalytic experiments, was also supplied from Sigma-Aldrich.

### 2.2. Synthesis of Bi_2_O_3_

Bi_2_O_3_ was synthesized following the method described in the literature with some modifications [40]. In more detail, 5 mmol of Bi(NO_3_)_3_·5H_2_O was dissolved in 10 mL of 1.5 M HNO_3_. Then, 10 mL of 4 M citric acid was added dropwise to the mixture under vigorous magnetic stirring for 40 min and under heating (70 °C) until it gelled. The obtained gel was dried at 80 °C overnight, then calcinated at 500 °C for 1h under air.

### 2.3. Preparation of PANI@Bi_2_O_3_-BiOCl Composite

PANI@ Bi_2_O_3_-BiOCl composites were prepared by in situ oxidative polymerization of aniline in the presence of Bi_2_O_3_ nanoparticles. First, a certain amount of Bi_2_O_3_ nanoparticles were dispersed in 100 mL of 1 M HCl by ultrasonic vibration for 3 h. Afterward, a specific amount of aniline was introduced into the mixture and stayed under stirring for 1 h. Next, dropwise to the reaction mixture was added 50 mL of Na_2_S_2_O_8_ (with a molar ratio aniline/oxidant of 1:2). The polymerization was maintained for 12 h under stirring at room temperature. The resultant powder was washed in abundance with distilled water and ethanol to eliminate oligoanilines and the oxidant excess. Finally, the residue (PANI@ Bi_2_O_3_-BiOCl) was kept for 12 h in the dryer at 80 °C.

Different initial weight ratios of aniline/Bi_2_O_3_ (from 0.2 to 5 wt.%) were tested in the experiments. For this reason, a series of PANI@Bi_2_O_3_-BiOCl composites having initial molar ratios of aniline/Bi_2_O_3_ 0.2, 0.5, 1, 2, and 5 wt.% were synthesized and labeled as 0.2 PBB, 0.5 PBB, 1PBB, 2PBB, and 5 PBB, respectively.

Furthermore, the Bi_2_O_3_-BiOCl composite was prepared by dispersing Bi_2_O_3_ nanoparticles in 100 mL of 1M HCl and kept under ultrasonic vibration for 3 h. Then the solution was filtrated, and the residue was washed with water and ethanol. The obtained powder was dried overnight at 80 °C.

### 2.4. Characterization

X-ray diffraction (XRD) analyses were performed using a PANanalytical ‘X’Pert PRO X-ray diffractometer with Cu Kα radiation and at 40 kV × 40 mA nominal X-ray power to characterize the phase(s) present in the resulting powders. The diffraction angles (2θ) were from 10° to 80°. The identification of the peaks from their positions and intensity was completed using the Joint Committee on Powder Diffraction Standards (JCPDS) database.

Fourier transform infrared spectroscopy (FTIR, Mattson 1000 FTIR spectrophotometer) was employed to investigate the vibration modes (ν, in cm^−1^), dispersing the proper amount of each sample in KBr and pressing the mixture to produce disks. The scans were taken in transmission mode in a wavenumber range of 4000–500 cm^−1^ and with a resolution of 4 cm^−1^. The samples morphology was investigated by scanning electron microscopy (SEM), performed on a Zeiss LEO 1525 field emission microscope equipped with an Inlens detector, upon metallization with chromium and an Energy Dispersive X-ray Analyzer (EDX) BRUKER.

Transmission electron microscopy (TEM) images were compiled by a Philips 208 transmission electron microscope (FEI, Hillsboro, OR, USA). The samples were prepared by depositing a small drop of the aqueous dispersion of the solids on a copper grid precoated with a Formvar film and then evaporated in the air at room temperature.

The obtained composites’ light absorption capacity and optical properties were quantified via UV–Vis spectrophotometer (Varian-Cary 100) equipped with an integrating sphere and using BaSO_4_ as a reference. The optical bandgap energy was calculated using the Kubelka-Munk function with Equation (1). [41]

A (hν − E_g_)*^n^*^/2^ = αhν (1)

where h is Planck’s constant, α is absorbance, ν is light frequency, and A is the proportional constant. The parameter n differs on the semiconductor properties, *n* = 1 and *n* = 4 for direct and indirect bandgap, respectively. BiOCl has an indirect bandgap, so *n* = 4, and α-Bi_2_O_3_ has a direct bandgap, so *n* = 1.

M-probe apparatus (Surface Science Instruments, Pasadena, CA, USA) was adopted for X-ray photoelectron spectroscopy (XPS) analyses: the source was a monochromatic Al Kα radiation (hν = 1486.6 eV). The precision of the stated binding energies (B.E.) can be approximated as ± 0.2 eV. Peak fitting was performed via combined Gaussian−Lorentzian curves after correcting the background using the Shirley method. The binding energies were calibrated using C1s at 284.6 eV.

### 2.5. Photocatalytic Experiments

Bi_2_O_3_, Bi_2_O_3_-BiOCl, and PBB nanopowders’ photocatalytic performance was estimated by the degradation of MB under solar light illumination. Before irradiation, catalysts and the aqueous solution of MB were stirred in the dark for 20 min to favor the adsorption-desorption equilibrium. The length of the photocatalytic experiments was 120 min. At a specific time, a volume of the solution was taken and filtered using a filter (RC 0.45 µm). The suspension concentration was determined by measuring the prominent peak of MB in the visible range at λ = 664 nm. Photolysis tests under illumination and without a catalyst were conducted. Under the experimental conditions applied in this work, substrate photolysis was unimportant.

### 2.6. Identification of Active Species

Identifying the active species responsible for photocatalytic activity was performed via Radical species trapping tests. Formic acid (CH_2_O_2_, 3 mM), benzoquinone (BQ, 7.65 μM), and isopropyl alcohol (C_3_H_8_O, 3 mM) were used as the scavengers for holes (h^+^), superoxide radicals (^●^O^2−^), and hydroxyl radicals (OH^●^), respectively [38,42]. 1 mmol of CH_2_O_2_, BQ, and C_3_H_8_O was added into a solution of MB containing 0.5 PBB nanocomposite, and the tests were conducted as described above (2.5).

## 3. Results

PANI/Bi_2_O_3_ composites have been previously synthesized by other researchers [39]. Their investigations revealed only Bi(III) species as Bi_2_O_3_. However, our studies demonstrate that during the oxidative polymerization reaction, a second Bi(III) component is obtained in the composite, identified as BiOCl, leading to ternary nanocomposites characterized by enhanced photocatalytic properties, as described below.

### 3.1. Characterization

**Figure 1:**
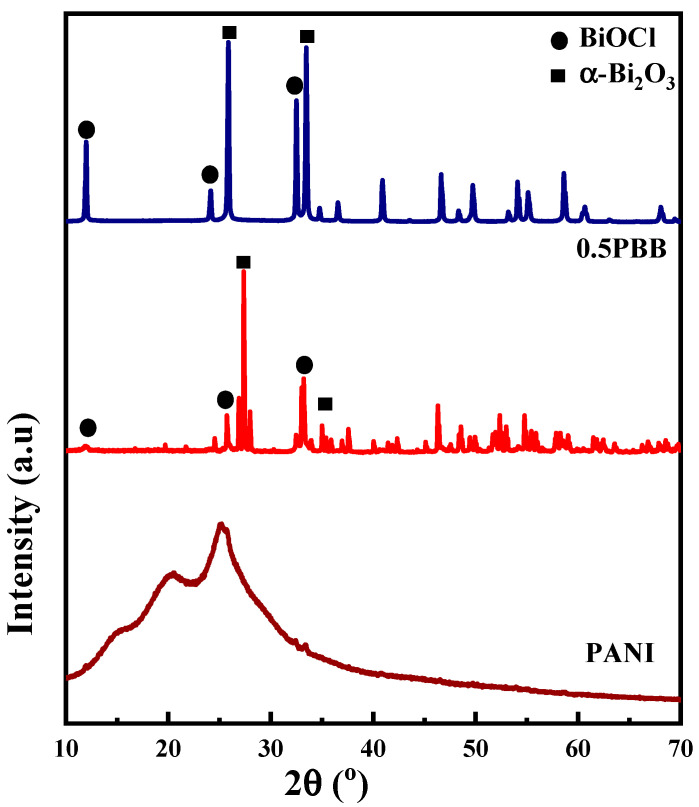
XRD patterns of PANI, Bi_2_O_3_-BiOCl, and 0.5PBB.

Figure 1 shows the crystal structure of the synthesized PANI, Bi_2_O_3_, Bi_2_O_3_-BiOCl, and 0.5PBB materials investigated by XRD analysis. Pristine PANI displays an XRD pattern characterized by two low diffraction peaks at 2θ = 20° and 25° that can be allocated to the periodicity perpendicular and parallel to the polymer chain [31]. The XRD pattern corresponding to α-Bi_2_O_3_ exhibits characteristic diffraction peaks at 27° and 33° corresponding to the diffractions of the (111) and (121) plane belonging to the monoclinic phase [43] (JCPDS No.41-1449) (Figure S1). However, the XRD pattern of 0.5PBB showed diffraction peaks corresponding to two different Bi(III) species, one corresponding to α-Bi_2_O_3_ and another one, characterized by a higher intensity of the diffraction peaks, identified as BiOCl tetragonal phase. The crystalline peaks attributed to BiOCl are slightly shifted to lower angles compared to pristine BiOCl. This shift can be related to the chemical interaction between PANI and Bi_2_O_3_-BiOCl composite, as generally observed for this type of heterostructures [44,45]. The quantitative analysis showed 87% α-Bi_2_O_3_ and 13% BiOCl in the 0.5PBB composite. In fact, during the oxidative polymerization step, Bi_2_O_3_ is dispersed in HCl solution, leading to the formation of BiOCl, according to the literature [46,47]. Figure 1 reports the diffraction pattern of Bi_2_O_3_ treated with HCl. It confirms that in addition to the monoclinic phases of Bi_2_O_3,_ other diffraction peaks are present at 12.0°, 23.9° and 33.4°, corresponding to the (0 0 1), (0 0 2) and (1 0 2) plan of the BiOCl tetragonal phase (JCPDS, no. 06-0249) [43]. These results confirmed the formation of BiOCl when Bi_2_O_3_ was dispersed in HCl, resulting in the obtention of the Bi_2_O_3_-BiOCl heterostructure.

**Figure 2:**
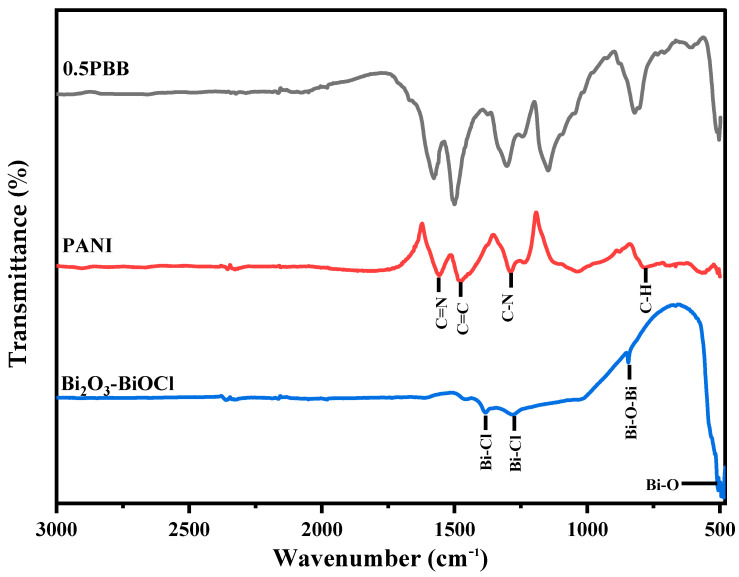
FTIR spectra of Bi_2_O_3_-BiOCl, PANI, and 0.5PBB.

The efficacious insertion of Bi_2_O_3_-BiOCl into the PANI matrix was also determined via FTIR. Figure 2 shows the acquired spectra of Bi_2_O_3_-BiOCl, PANI, and 0.5PBB. Typical bands at 1571 cm^−1^ and 1465 cm^−1^ ascribed to C=N and C=C stretching vibrational modes of quinoid and benzenoid rings were recorded in the PANI’s spectrum [48,49]. Likewise, the 1271 cm^−1^ band is ascribed to the C-N benzenoid stretching, while the 805 cm^−1^ is assigned to the out-of-plane deformation of C-H in the benzene ring [50]. The presence of the benzenoid and quinoid rings implies that PANI was obtained in its emeraldine form (half-oxidized) [51]. The FTIR spectrum of Bi_2_O_3_-BiOCl showed typical bands at 847 cm^−1^ and 494 cm^−1^, ascribed to Bi-O-Bi and Bi-O stretching, respectively. The band at 847 cm^−1^ is characteristic of Bi_2_O_3_ and related to the Bi-O-Bi vibration mode (Figure S2). Moreover, the bands at 1035 and 1103 cm^−1^ can be ascribed to the symmetric and asymmetric stretching vibrations of Bi-Cl. The band at 1382 cm^−1^ is assigned to the NO^3−^ group [52]. The presence of NO^3−^ indicates a potential competition of NO^3−^ with Cl^−^ in emeraldine salt structure [53]. After HCl treatment, the main band of Bi_2_O_3_ decreased, confirming the growth of BiOCl on Bi_2_O_3_ to construct the heterostructure, according to the literature [54]. The FTIR spectrum of the 0.5PBB nanocomposite exhibits the characteristic bands of both the components, organic and inorganic. The high intensity of the signals related to the polymeric components partially covers those of Bi_2_O_3_-BiOCl, although the typical band at 1143 cm^−1^ related to the Bi-Cl stretching vibration is well evident [55]. Eventually, the slight shift of the bands of the PANI/Bi-based materials confirms the fruitful interaction between the two components of the nanocomposite, in agreement with the XRD results.

**Figure 3:**
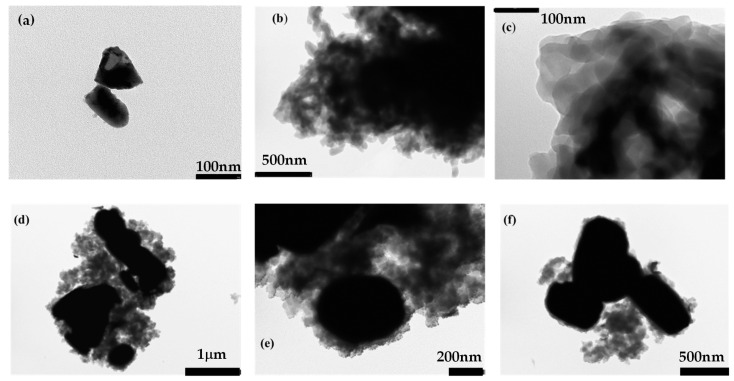
TEM Images of (**a**) Bi_2_O_3_, (**b**) and (**c**) PANI, (**d**–**f**) 0.5PBB composite.

**Figure 4:**
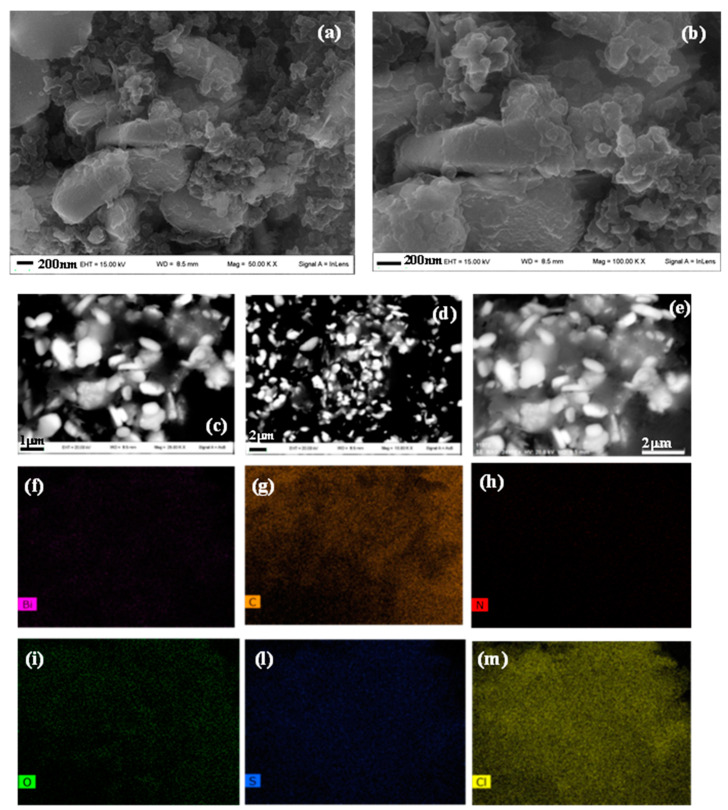
SEM images of 0.5PBB (**a**–**e**) and elemental mapping of (**f**): Bi, (**g**): C, (**h**): N, (**i**): O, (**l**): S, (**m**): Cl for 0.5PBB composite.

SEM and TEM techniques examined the morphologies of PANI, Bi_2_O_3,_ and 0.5PBB. The obtained images are shown in Figure 3, Figure 4, Figures S3 and S4 (in the SI). The TEM and SEM images of Bi_2_O_3_ exhibited a spindle-like shape of about 100 nm in size, as shown in Figure 3a and Figure S3a–c. Moreover, the EDS analysis and the O and Bi elemental mapping of Bi_2_O_3_ are reported in Figure S3d–f. The TEM image of PANI (Figure 3b,c) showed the typical globular-like morphology, a characteristic of this polymer, as previously observed [22].

According to the results of XRD and FTIR investigations, TEM images of 0.5PBB nanocomposite showed nanocomposites containing Bi_2_O_3_ -BiOCl particles (dark objects) embedded into PANI matrix (Figure 3d–f) [56]. Besides, the SEM images of PANI and the 0.5PBB nanocomposite (Figure S4a,b,d and Figure 4a,b) were acquired, as well as EDS analyses and elemental mapping were properly performed (Figures S4c–h and S5, and Figure 4f–m). For the composite, the SEM images were also carried out by an angle-selective backscatter detector (Figure 4c,d), by which elements having higher atomic numbers emit backscattered electrons with greater intensity. These images show that the Bi_2_O_3_ morphology changes upon insertion into the PANI, forming nanosheets homogenously distributed in the polymeric matrix. According to the literature, the nanosheet morphology can be attributed to the BiOCl formation [56,57]. The EDS spectrum (Figure S5) and the elemental mapping (Figure 4f–m) of 0.5PBB prove the presence and homogenous distribution of Bi, O, C, and N. Furthermore, the presence of Cl can confirm the formation of the BiOCl, even if it can also be related to the use of the usual HCl dopant.

**Figure 5:**
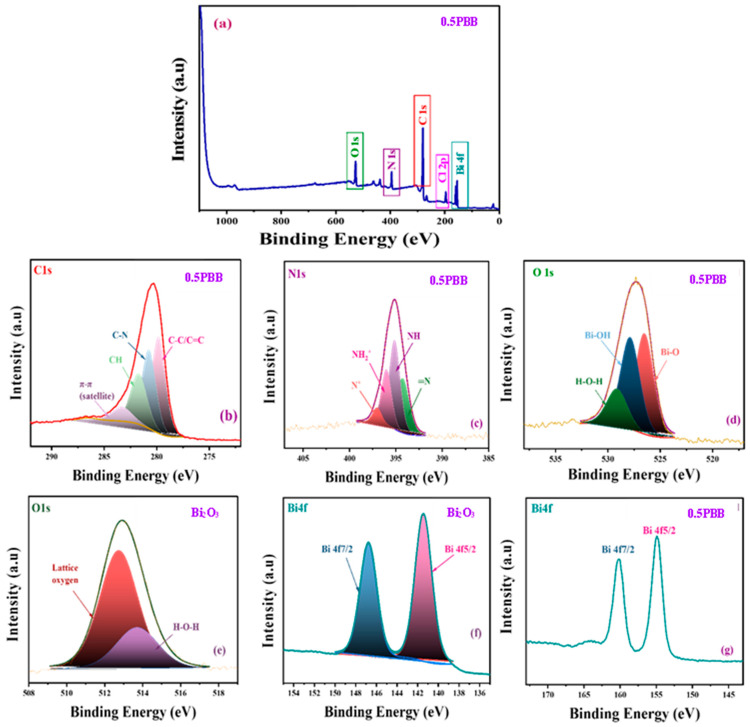
XPS results of (**a**) survey spectra of 0.5PBB. HR spectra of (**b**) C 1s, (**c**), N1s, (**d**,**e**) O1s, and (**f**,**g**) Bi4f of Bi_2_O_3_ and 0.5PBB composite.

XPS was used to investigate the surface element composition and chemical state of 0.5PBB and Bi_2_O_3_. The XPS survey of 0.5PBB (Figure 5a) exhibits peaks related to Bi and O coming from Bi_2_O_3_ and BiOCl, as well as C and N from PANI. In addition, the peak of Cl can be ascribed to the presence of both BiOCl and PANI. The XPS survey of Bi_2_O_3_ is reported in Figure S6. The high-resolution XPS spectra of C1s, O1s, N1s, and Bi4f are shown in Figure 5b–g. The deconvolution of the High-Resolution spectrum of C1s (Figure 5b) gave several parts at 284.6, 285.6, 287.3, and 291.5 eV assigned to C-C/C=C, C-N, CH, and π-π (satellite) respectively [48,58]. Figure 5c shows the HR XPS spectrum of N1s, which can be curve-fitted into four peaks related to the presence of neutral imine nitrogen atom, neutral amine, positively charged nitrogen, and protonated imines belonging to the PANI structure [48]. The core-level spectrum fitting to O1s can be deconvoluted into two peaks (Figure 5d) at 529.7 eV and 531.3 eV assigned to Bi-O in Bi_2_O_3_ and a peak at 533.5 eV associated with physisorbed and chemisorbed water and other surface species such as coordinated lattice oxygen, OH, and O_2_ chemisorbed [41]. However, the O1s core spectrum of Bi_2_O_3_ displays only chemisorbed or physisorbed water and oxygen functionalities (Figure 5e). The HR XPS spectrum of Bi (Figure 5f,g) can be deconvoluted into two peaks at 159.04 eV and 164.3 eV corresponding to the signals from doublets of Bi 4f5/2 and Bi 4f7/2 in the trivalent oxidation state. Compared to the pristine Bi_2_O_3_ spectrum, it is evident that the peaks belonging to Bi4f of 0.5PBB shifted to higher energy, which confirms that the combination of PANI impacts the electronic energy distribution of Bi4f via the interfacial coupling interaction. The raised binding energy may be essential in effectively separating charges [59].

**Figure 6:**
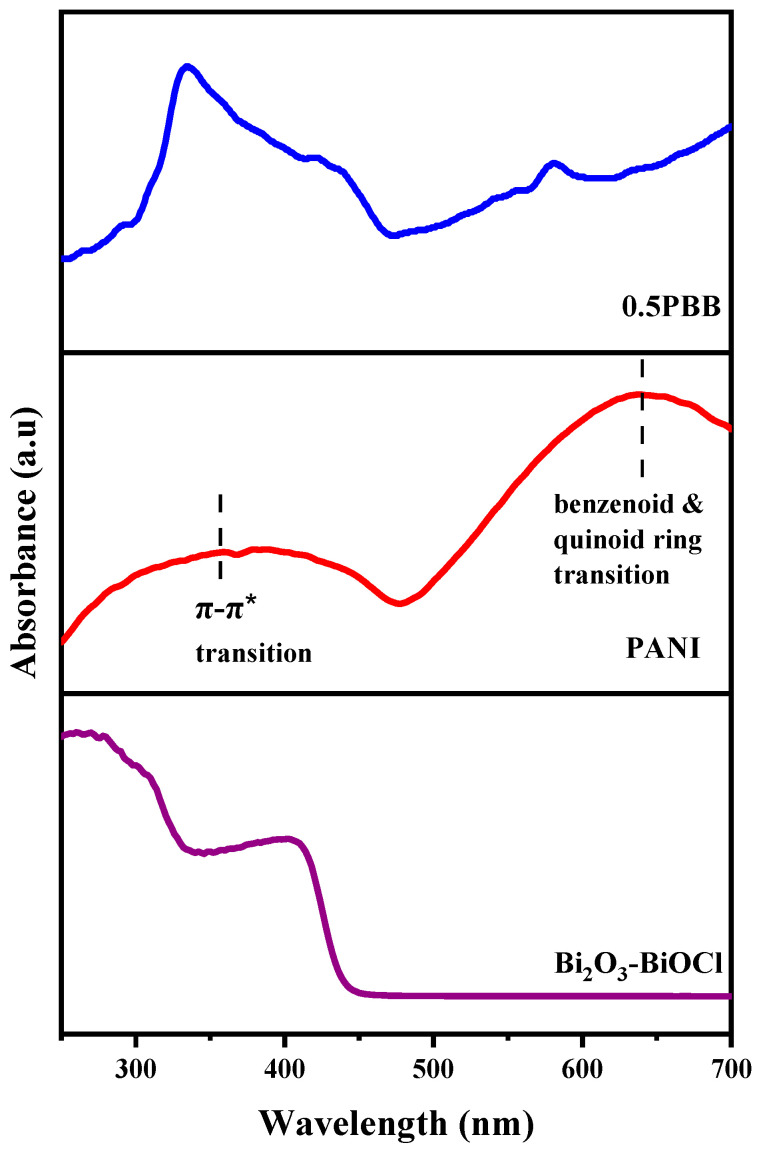
UV–Visible spectra of Bi_2_O_3_-BiOCl, PANI, and 0.5PBB.

The obtained materials’ spectral characteristic and optical properties were investigated using UV–Vis diffuse reflectance spectra (DRS). The spectrum corresponding to α-Bi_2_O_3_ (Figure S7) displays a strong visible-light absorption, and the adsorption edge is near 411nm. In contrast, as expected, the spectrum of the Bi_2_O_3_-BiOCl heterostructure exhibits two absorption bands, one in the UV range (due to the presence of BiOCl) and the second one in the visible light range (Figure 6). On the other hand, the spectrum of PANI displayed 2 distinctive absorption bands, one in the near-infrared (around 350 nm) belonging to π–π* transition on the benzenoid ring and the other broad peak in the visible region between 500–700 nm assigned to the excitation transition of the benzenoid and quinoid ring (Figure 6) [30,60]. It is evident that the presence of PANI affects the light absorption characteristic of α-Bi_2_O_3_-BiOCl nanopowder, and the obtained composite displays a combined ability of absorption belonging to the two components. Hence, the synthesized composite gained a considerably improved absorption at the visible range compared to the pristine components, confirming the successful combination of PANI and Bi_2_O_3_-BiOCl.

The bandgap energy of α-Bi_2_O_3_, Bi_2_O_3_-BiOCl, PANI, and 0.5PBB was calculated, and the values were 2.80 eV, 2.70 eV, 2.80 eV, and 2.52 eV, respectively. These results demonstrate that adding 0.5% of PANI resulted in narrow bandgap energy compared with pristine Bi_2_O_3_, PANI, and Bi_2_O_3_-BiOCl composite due to the hybrid’s strong interaction resulting in a more apparent use of solar spectrum. Likewise, the fruitful combination of the π-conjugated structure of PANI with the Bi_2_O_3_-BiOCl heterostructure should result in an easy photoinduced charges migration and charge recombination restriction.

The specific surface area of Bi_2_O_3_-BiOCl, 0.2PBB,0.5PBB, 1PBB, 2PBB, and 5PBB composites were investigated via BET adsorption/desorption of nitrogen. Concerning Bi_2_O_3_ and Bi_2_O_3_-BiOCl, the specific surface area was 28.0 m^2^/g and 38.2 m^2^/g, respectively. The rise in the specific surface area after dispersing Bi_2_O_3_ in HCl could be related to the formation of BiOCl nanosheets. However, as for PBB nanocomposites, the specific surface area decreased as the PANI amount increased, and the maximum surface area was obtained for 0.2PBB composite (16.5 m^2^/g). In fact, 0.5PBB, 1PBB, 2PBB, and 5PBB displayed specific surface areas of 11.6 m^2^/g, 8.5 m^2^/g, 6.8 m^2^/g, and 6.7m^2^/g, respectively. The findings are in agreement with earlier published work [31].

**Figure 7:**
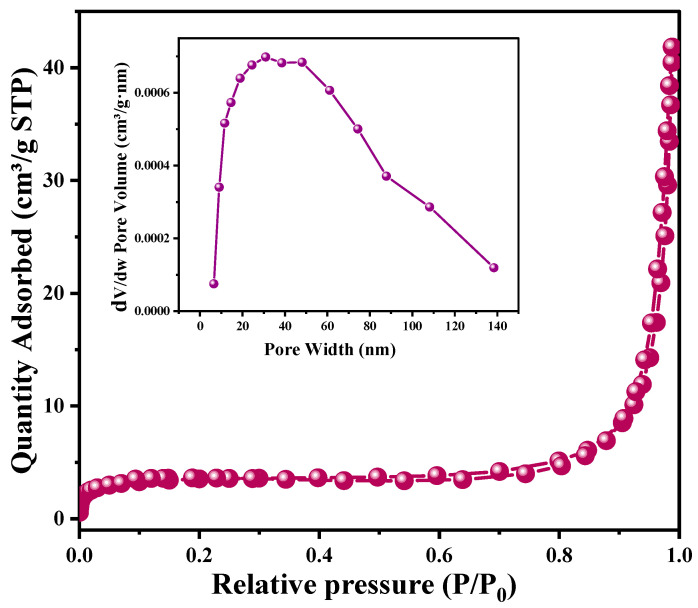
N_2_ adsorption/desorption isotherm of 0.5PBB composite.

The obtained isotherm belonging to 0.5PBB is given in Figure 7. 0.5PBB nanocomposite showed monolayer nitrogen adsorption at low relative pressures, where P/P_0_ is less than 0.1 and a relatively small hysteresis loop at a relative pressure between 0.45–0.9. Based on the IUPAC standard classification, the N_2_ adsorption/desorption isotherm is of Type IV, indicating that the structure of 0.5PBB is mesoporous.

### 3.2. Photocatalytic Test

**Figure 8:**
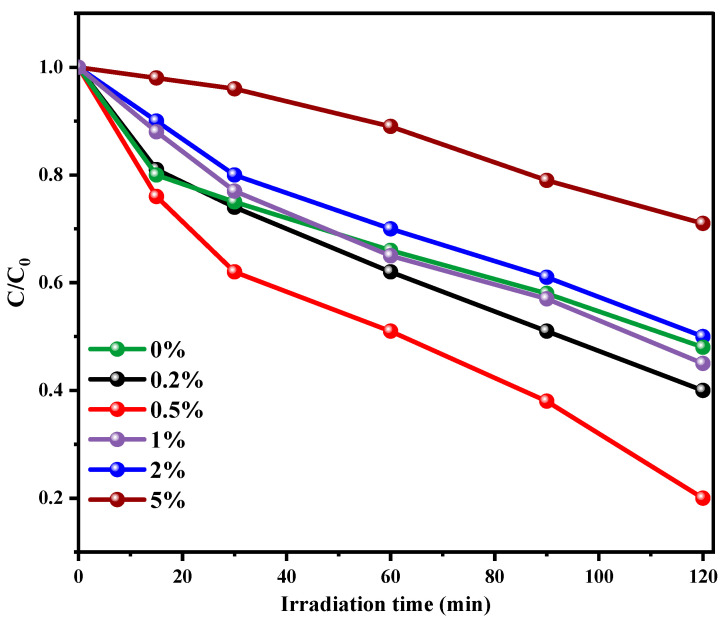
Conversion plots for the MB photodegradation for synthesized composite under solar light irradiation.

**Figure 9:**
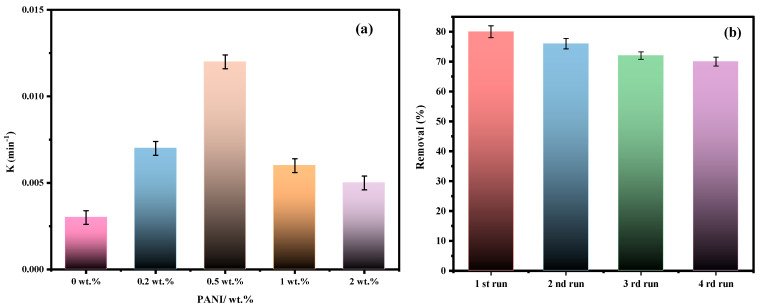
(**a**) Rate constant of Bi_2_O_3_-BiOCl and PBB composites. (**b**) Reuse of 0.5PBB for the photocatalytic degradation of MB under solar light illumination.

The photocatalytic performance of the materials was studied by following the photodegradation of MB under solar light irradiation at ambient temperature. The photodegradation efficacy in the absence of any material (photolysis) and the presence of α-Bi_2_O_3_ nanopowder and PBB nanocomposites are displayed in Figure S8. In the absence of the photocatalyst, the photodegradation is negligible, as well as when the reaction is carried out in the dark. As expected, PANI shows no activity towards MB removal under visible light. In fact, the abatement of MB reached only 20% after 120 min (Figure S8). The Bi_2_O_3_ nanopowder displayed 38% degradation after 120 min irradiation (Figure S8). These results agree with the previous reports [61]. Combining Bi_2_O_3_-BiOCl with PANI results in an apparent enhancement in photocatalytic activity. The found photodegradation efficiency for 0.2PBB, 0.5PBB, 1PBB, 2PBB, and 5PBB was 60%, 80%, 55%, 50%, and 29%, respectively (Figure 8). From these outcomes, it can be assumed that the PANI amount added is a crucial factor affecting photocatalytic degradation efficacy. Inserting PANI into Bi_2_O_3_-BiOCl enhances the photocatalytic efficiency; however, too much PANI has the opposite impact. As reported above, the maximum photodegradation activity was obtained for 0.5PBB nanocomposite (Figure 8), reaching an MB degradation of 80% after 2 h. It is evident that a good dispersion of PANI in Bi_2_O_3_-BiOCl is related to the amount of PANI added, which leads to the transfer and separation of charge carriers while conserving the surface availability for oxidation. In contrast, if the amount of PANI exceeds a certain amount, it may limit contact with the oxidation site due to the surface coverage [52]. Plotting ln(C/C_0_) versus illumination time (Figure S9) linear curves were obtained, suggesting that the MB photodegradation by PBB is a pseudo-first-order reaction. Figure 9a displays the apparent constant rate (k) for the composites having various PANI amounts. The maximum K value was 0.012 min^−1^ for 0.5 PBB, which is four times higher than that of pristine Bi_2_O_3_-BiOCl (0.003 min^−1^), confirming the fruitful interaction between PANI and Bi_2_O_3_-BiOCl, leading to enhanced photocatalytic activity. The catalytic activity of the ternary PANI@Bi_2_O_3_-BiOCl reported in this study was better than the one reported when PANI@Bi_2_O_3_ and PANI@BiOCl were used [37,38].

The photocatalyst re-usage for several runs is a vital factor for applications. For this purpose, the 0.5PBB nanocomposite was analyzed for 4 cycle runs. At the end of each cycle, the catalyst was retrieved via centrifugation and immediately reused without any treatment. Figure 9b displays the obtained results. It is worth stating that the 0.5PBBB catalyst showed good stability up to four cycles; however, it exhibited a gradual slow decrease in photoactivity. Starting from an initial 80% of photodegradation at the photoactivity dropped to 70% during the fourth cycle, which could be due to nanoparticles’ aggregation and/or irreversible adsorption of the dye transformation product on the surface of the polymeric matrix leading to the blockage of photocatalytic active sites.

### 3.3. Identification of Active Species

**Figure 10:**
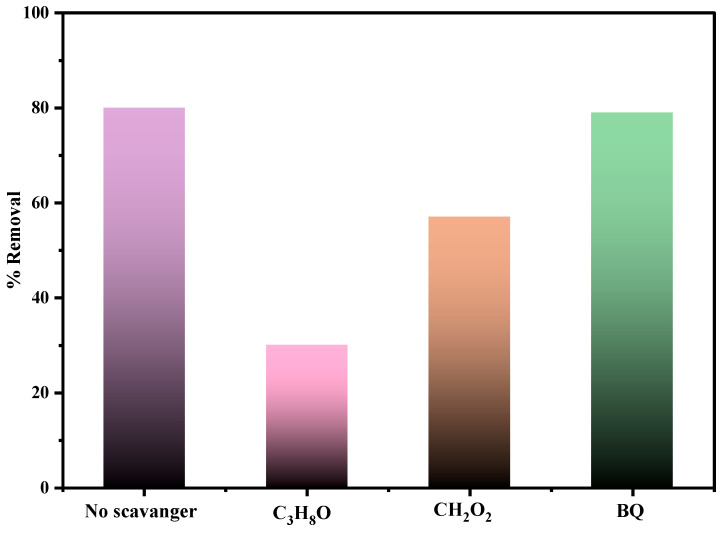
MB Photocatalytic degradation by 0.5PBB composite upon solar light irradiation in the presence of scavengers (C_3_H_8_, CH_2_O_2_, and BQ).

Numerous radical trapping tests for MB degradation in the presence of 0.5PBB under solar light irradiation were carried out to understand the photocatalytic mechanism occurring on the PBB composites. C_3_H_8_O, CH_2_O_2_, and BQ were used to trap OH^●^, h+, and ^●^O^2−^, respectively. Figure 10 shows that the addition of C_3_H_8_O caused a reduction in the photocatalytic efficiency from 80% to 30%. These findings demonstrate the key role of OH^●^ in the photocatalytic process. Similarly, a considerable decrease in the photocatalytic activity was observed from 80% to 57% after adding CH_2_O_2_, meaning h^+^ is another major oxidizing species. On the contrary, upon adding BQ, the efficiency showed a negligible drop, implying that ^●^O^2−^ is not an active radical specie in the MB photodegradation. It can be assumed from these outcomes that the active species influencing most of the photo-oxidative degradation of MB by 0.5 PBB are OH^●^ and h^+^. Other researchers reported similar results [38,46].

### 3.4. Photocatalytic Mechanism

**Figure 11:**
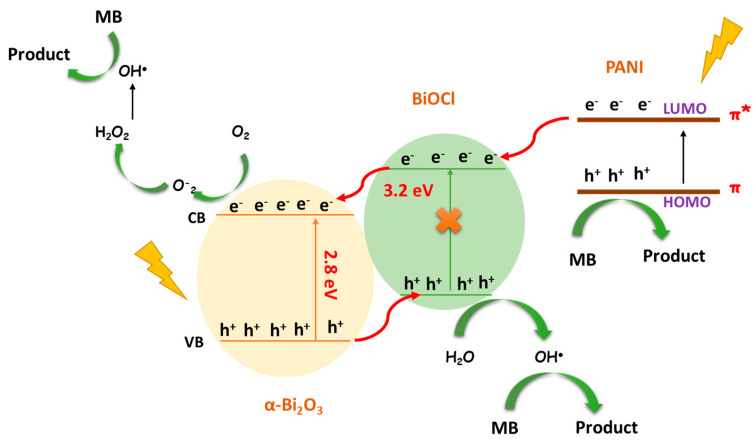
Schematic photocatalytic mechanism of photocatalyst PB composite.

The photocatalytic results show that the coating of Bi_2_O_3_-BiOCl with PANI led to increased photocatalytic efficiency. However, increasing the amount of PANI above 0.5% (wt.) caused a reduction in the photocatalytic degradation of MB, presumably due to excess PANI blocking contact with the oxidation site because of the surface coverage [6]. The photo-oxidation of the species absorbed at the surface of Bi_2_O_3_-BiOCl is limited by the position of the conduction band minimum (CBM) and valence band maximum (VBM). To examine the effect of PANI addition on the PBB bandgap structure, the CBM and VBM were determined theoretically, and the obtained values are presented in Figure 11. The conduction band (Ec) energy was calculated using Equation (2) [62,63].

E_c_ = X − E + 0.5E_g_ (2)

E_c_: semiconductor edge conduction band at 0 charge point, X: absolute electronegativity of semiconductor (Mulliken) (X = 5.95 eV and 5.0 eV for Bi_2_O_3_ and BiOCl, respectively), computed from the electronegativity absolute geometric mean of the constituent atoms, defined as the atomic electronic affinity arithmetic means and the energy of the first ionization. E is the energy of free electrons on the hydrogen scale (~4.5 eV), and E_g_ is the bandgap energy of the semiconductor.

The Eg of α-Bi_2_O_3_, BiOCl, and PANI are 2.8, 3.21 [22], and 2.8 eV, respectively. The conduction band potentials for α-Bi_2_O_3_ and BiOCl are +0.21 and −1.11 eV, and the valence band potentials are +3.01 and +2.10 eV, respectively. Whereas HOMO and LUMO of PANI are 0.8 eV and −1.9 eV (vs. NHE) [64].

The visible illumination efficiently generates electron transition from VB to the CB for samples with low bandgap values, causing an equal amount of unoccupied sites. In addition, a fraction of electron-hole (e^−^/h^+^) pairs recombine, and the input energy is emitted as heat or light. This recombination is balanced by an electron transition from VB to CB. In order to improve the photocatalytic activity, the optimization of electron transition and the e^−^/h^+^ pairs must be performed. Thus, the photocatalyst with the appropriate Eg displayed an enhanced degradation rate.

Theoretically and based on the heterostructure transfer of photogenerated carriers, PANI can inject electrons into the CB of BiOCl. Then, the holes present at the VB of Bi_2_O_3_ can be transferred to the VB of BiOCl via the heterostructure formed, leading to an efficient e^−^/h^+^ separation [34,61]. However, the CB of Bi_2_O_3_ is less negative (+0.21 eV) than the O_2_/O_2_^•−^ potential (−0.33 eV vs. NHE), signifying the photogenerated electrons at the Bi_2_O_3_ CB are incapable of reducing O_2_ and generating O_2_^•−^ [38]. These findings confirm the results of the active species trapping investigation, which stated that O_2_^•−^ does not take part in the photodegradation of MB.

Based on the findings obtained from radical scavenger experiments, a mechanism for the photocatalytic performance was given in Figure 11. Under sun-illumination, PANI and Bi_2_O_3_ are excited due to their low bandgap energy, and the photoelectrons and holes are formed. Since the LUMO potential of PANI is more negative than the CB of BiOCl, the photoinduced electrons of PANI will migrate into the CB of BiOCl [6,65]. Likewise, since the CB of BiOCl is more negative than that of α-Bi_2_O_3_, the e^−^ on the BiOCl surface can easily migrate to the α-Bi_2_O_3_ and generate OH^●^ from H_2_O_2_. Moreover, because of the VB position difference between Bi_2_O_3_ and BiOCl, h^+^ generated on the VB of the α-Bi_2_O_3_ will also be transferred to the VB of BiOCl, forming an internal electric field across the interface. Thus, the delay of the e^−^/h^+^ recombination is due to the efficiency of photogenerated electrons and hole separation, which is crucial for enhancing photocatalytic activity. The charge separation efficiently lowers the recombination rate of electron/hole, thus improving the photocatalytic performance at the ternary α-Bi_2_O_3_@BiOCl@ PANI composite. According to the experimental outcomes, the plausible photocatalytic mechanisms are given in Equations (3)–(6):

(3) $$\mathrm{PANI}@{\mathrm{Bi}}_{2}O_{3}-\mathrm{BiOCl}\;+\mathrm{hv}\;\to {\;h}^{+}+{\;e}^{-}\;$$

(4) $$H_{2}O^{-}+{\;h}^{+}\;\to {\;\mathrm{OH}}^{•}$$

(5) $$h^{+}+\mathrm{MB}\;\to \;\mathrm{Degradation}\;\mathrm{products}\;$$

(6) $${\mathrm{OH}}^{•}+\mathrm{MB}\;\to \;\mathrm{Degradation}\;\mathrm{products}\;$$

## 4. Conclusions

In summary, ternary polyaniline@Bi_2_O_3_-BiOCl nanocomposite photocatalysts with various amounts of PANI were efficiently obtained via in-situ polymerization of aniline in the presence of Bi_2_O_3_. XRD, FTIR, TEM, SEM-EDS, XPS, and DRS techniques were used to confirm the synthesis of composites. The third Bi(III) phase (BiOCl) was obtained during the in-situ polymerization step. The results show that the addition of PANI up to 1% increased the photocatalytic activity of the nanocomposite. Furthermore, 0.5PBB composite demonstrated the maximum photocatalytic degradation efficacy, degrading 80% of MB after two hours of solar light illumination. The enhancement in photocatalytic activity is associated with forming a heterostructure between α-Bi_2_O_3_, BiOCl, and PANI, promoting the photogenerated e−/h^+^ pairs separation. However, adding too much PANI had a negative effect due to limit contact with the oxidation site due to the surface coverage. The nanocomposite was stable for all the investigated cycles, always maintaining high MB photodegradation values. The scavenger test established that hole h^+^ and OH^●^ radicals are the active radical species in the photocatalytic process. The efficient charge separation enhanced interfacial charge transfer and the higher activity of the adsorption sites are primarily due to the positive synergetic effect between PANI and Bi_2_O_3_-BiOCl.

## Data Availability

Not applicable.

## Supplementary Materials

The following supporting information can be downloaded at: https://www.mdpi.com/article/10.3390/nano13040713/s1, Figure S1. XRD patterns of Bi_2_O_3_; Figure S2. FTIR spectrum of Bi_2_O_3_; Figure S3. (a) TEM image, (b,c) SEM images, (d,e) O and Bi elemental mapping, and (f) EDS spectrum of Bi_2_O_3_; Figure S4. (a,b) SEM Image, (c–g) elemental mapping and EDS spectrum PANI; Figure S5. EDS spectrum of 0.5PBB; Figure S6. XPS survey spectra of Bi_2_O_3_; Figure S7. UV-visible spectra of Bi_2_O_3_; Figure S8. Conversion plots for the photodegradation of MB by PANI, Bi_2_O_3_, blank and in Dark; Figure S9. Kinetic curves (ln(C/C_0_) = f(t)) of MB photodegradation.
