# Supplementary material for: Ternary Polyaniline@Bi2O3-BiOCl Nanocomposites as Innovative Highly Active Photocatalysts for the Removal of the Dye under Solar Light Irradiation
Source: Nanomaterials (Basel). 2023 Feb 13;13(4):713. doi: 10.3390/nano13040713 (PMC9961263; doi:10.3390/nano13040713)
Supplement: Supplementary file 1 [file nanomaterials-13-00713-s001.zip › nanomaterials-2221918-supplementary.pdf]

Supplementary Materials

Ternary Polyaniline@Bi_2O_3-BiOCl Nanocomposites as Innovative Highly Active Photocatalysts for the Removal of the Dye under Solar Light Irradiation

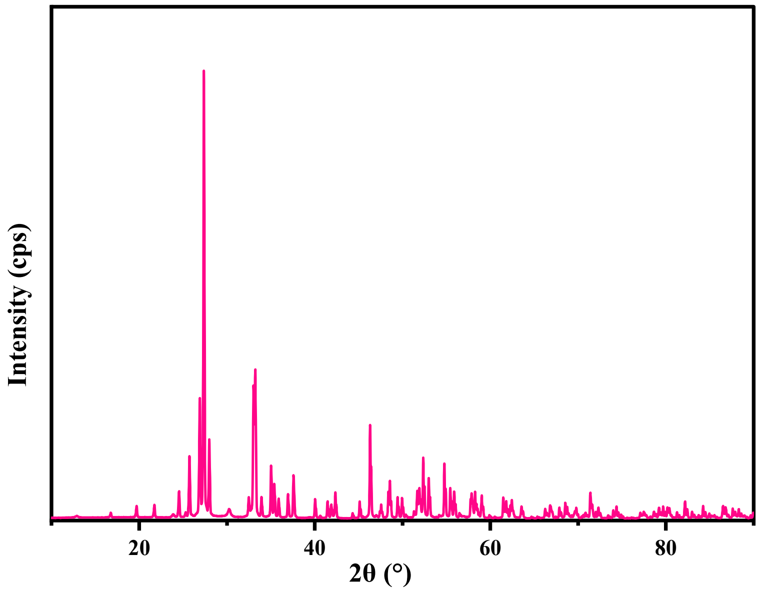

Figure S1. XRD patterns of Bi_2O_3

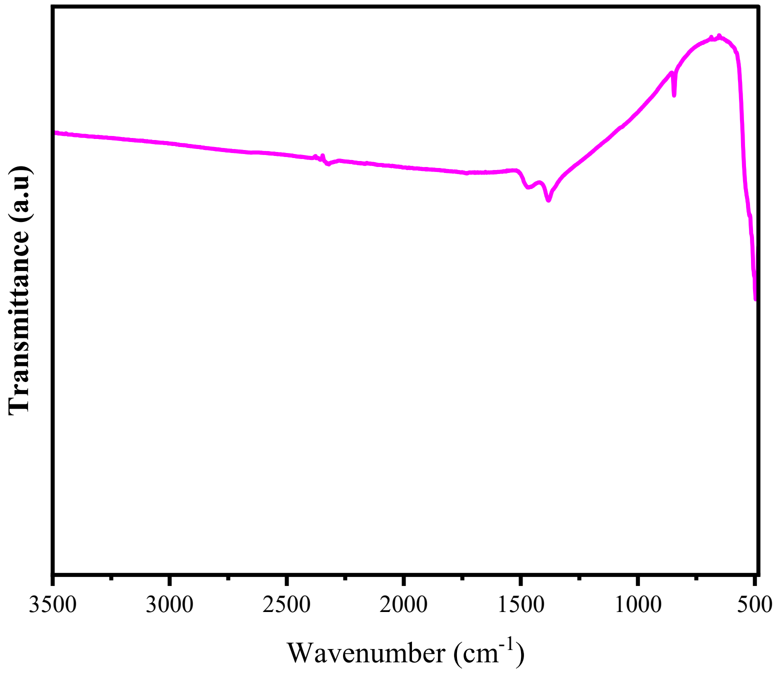

Figure S2. FTIR spectrum of Bi_2O_3

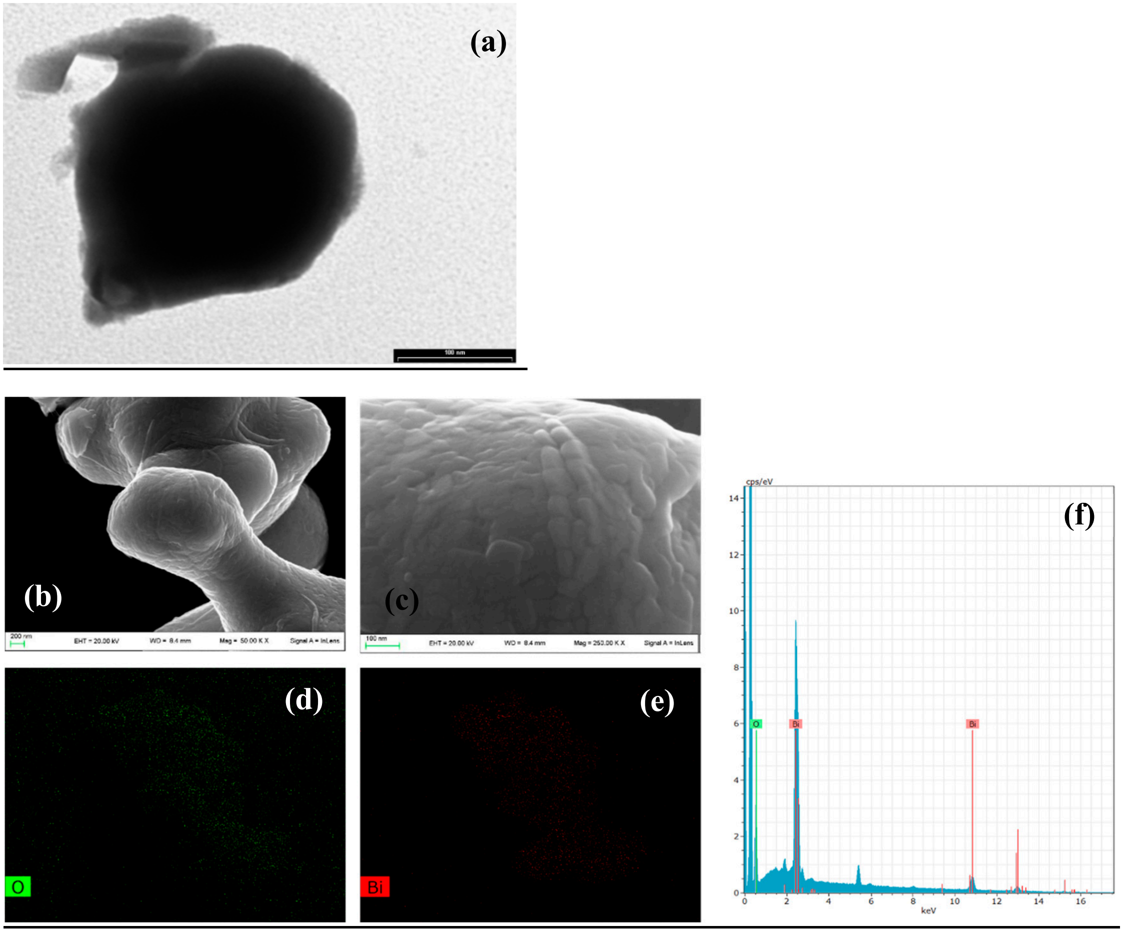

Figure S3. (a) TEM image, (b and c) SEM images, (d and e O and Bi elemental mapping and (f) EDS spectrum of Bi_2O_3

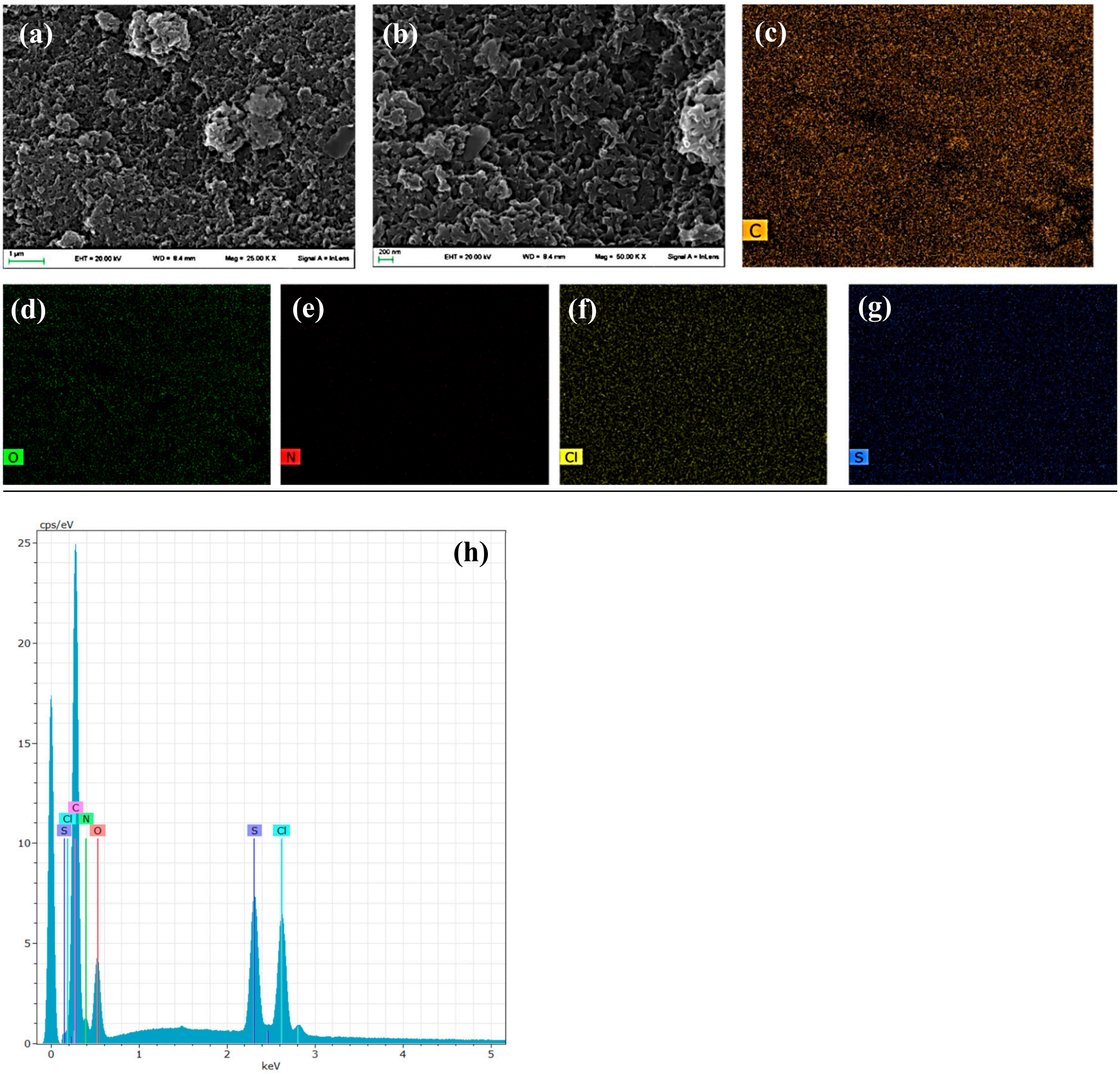

Figure S4. (a and b) SEM Image, (c-g) elemental mapping and EDS spectrum PANI

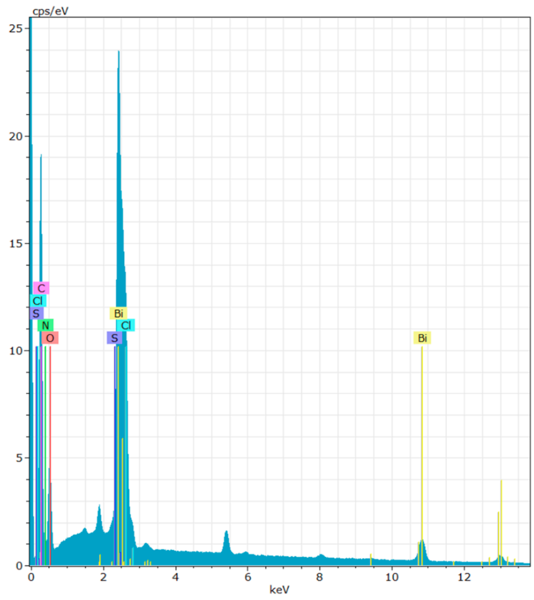

Figure S5. EDS spectrum of 0.5PBB

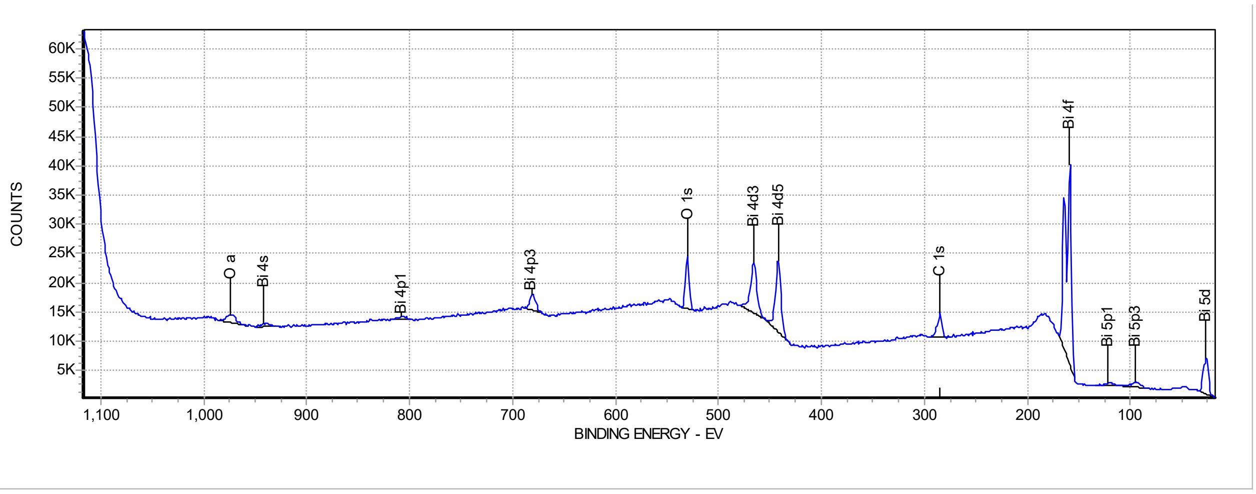

Figure S6. XPS survey spectra of Bi_2O_3

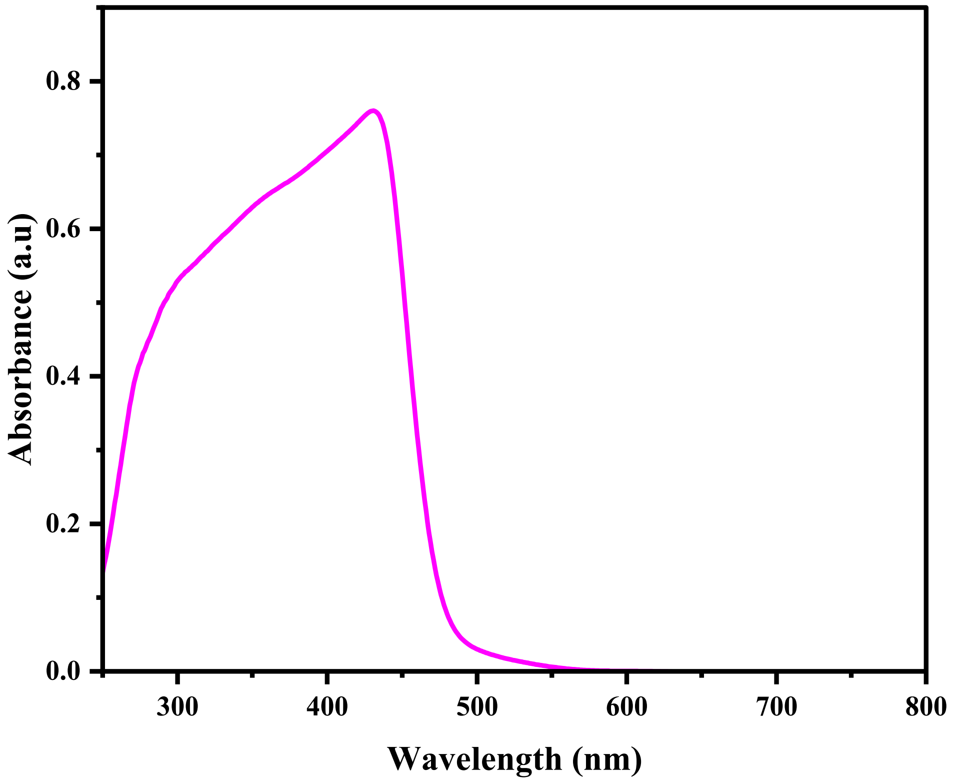

Figure S7. UV-visible spectra of Bi2O3

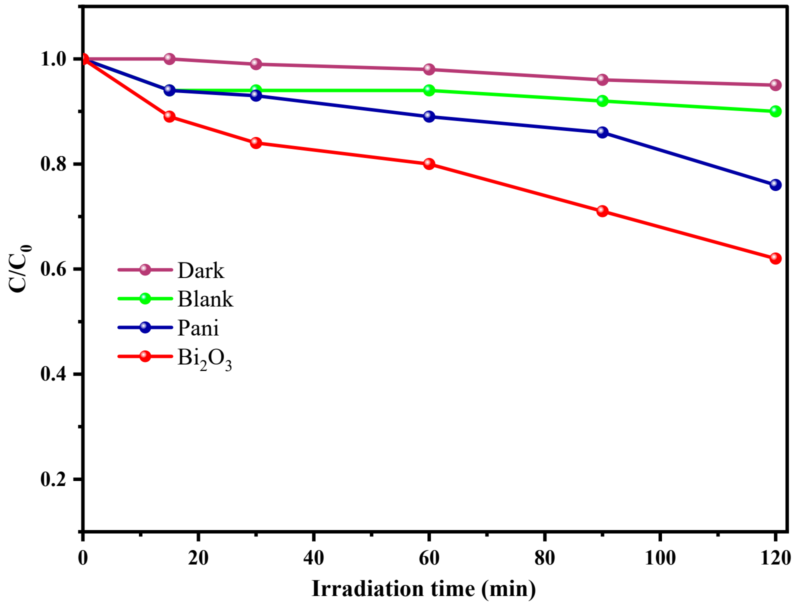

Figure S8. Conversion plots for the photodegradation of MB by PANI, Bi2O3, blank and in Dark

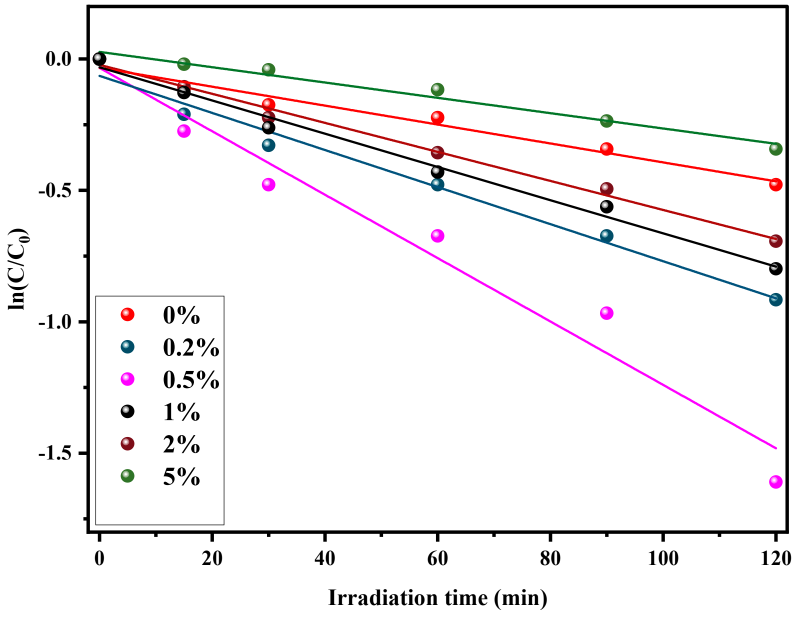

Figure S9. Kinetic curves ($ln(C/C_0)= f(t)$) of MB photodegradation
